# Health-Related Effects of Short Stays at Mountain Meadows, a River and an Urban Site—Results from a Field Experiment

**DOI:** 10.3390/ijerph15122647

**Published:** 2018-11-26

**Authors:** Arne Arnberger, Renate Eder, Brigitte Allex, Martin Ebenberger, Hans-Peter Hutter, Peter Wallner, Nicole Bauer, Johann G. Zaller, Thomas Frank

**Affiliations:** 1 Institute of Landscape Development, Recreation and Conservation Planning, Department of Spatial, Landscape and Infrastructural Sciences, University of Natural Resources and Life Sciences Vienna, Vienna 1190, Austria; renate.eder@boku.ac.at (R.E.); brigitte.allex@boku.ac.at (B.A.); martin.ebenberger@boku.ac.at (M.E.); 2 Department of Environmental Health, Center for Public Health, Medical University Vienna, Vienna 1090, Austria; hans-peter.hutter@meduniwien.ac.at (H.-P.H.); peter.wallner@meduniwien.ac.at (P.W.); 3 Swiss Federal Institute for Forest, Snow and Landscape Research (WSL), Economics and Social Sciences, Social Sciences in Landscape Research, Birmensdorf 8903, Switzerland; nicole.bauer@wsl.ch; 4 Institute of Zoology, University of Natural Resources and Life Sciences Vienna, Vienna 1180, Austria; johann.zaller@boku.ac.at (J.G.Z.); thomas.frank@boku.ac.at (T.F.)

**Keywords:** the Alps, pulse rate, blood pressure, naturalness, landscape perceptions, sound perceptions

## Abstract

The study compared psychological and physiological health effects of short-term stays at managed and abandoned meadows, a mountain river, and an urban site of a dependent sample of 22 adult participants (mean age 27) during an 11-day field trip. The study found that pulse rates decreased during the stays at all the meadows and the urban site while no decrease was observed at the river. Blood pressure increased at all sites during the stay, with no study-site differences for systolic, but for diastolic, blood pressure. Participants reported more positive psychological health effects as a result of their stays at the most remote meadow and the river on attention restoration, stress reduction and wellbeing compared to the urban site, while no differences in health perceptions were observed between managed and unmanaged meadows. This study suggests that perceived and measured health benefits were independent of the degree of naturalness of meadows. While differences measured on the physiological level between urban built and natural sites were marginal, psychological measures showed higher health benefits of the natural environments compared to the built one.

## 1. Introduction

Restorative research has found that exposure to natural and semi-natural environments provides more health benefits than exposure to urban built-up or street environments [1,2,3,4,5,6]. Although a substantial amount of evidence revealed the restorative potential of natural environments in general, little is known about the effects of specific landscape types on human health and wellbeing [1,7,8,9,10,11]. 

In particular, the role mountain landscapes including green and water-based environments play on human health has been little investigated. The question arises of whether mountain meadows or mountain rivers can provide health benefits to humans compared to urban environments, and whether there are differences in restorative effects between mountain meadows depending on their degree of naturalness.

### 1.1. Health Benefits of Green Environments on Humans

Two principal theories are used to explain landscape-based human health effects: the Stress Reduction Theory (SRT) [5] and the Attention Restoration Theory (ART) [6]. SRT [5] assumes that a visit to natural environments can assist in recovering from stress. Research findings confirmed that, compared to built-up settings, exposure to natural settings can lead to positive changes in mood and stress reduction and lower heartbeat rates [4,5,12]. Ulrich et al. [5], for example, demonstrated faster and more complete stress recovery in settings dominated by trees. ART asserts that people can concentrate better after a stay in nature compared to a stay in the built environment. Restoring people’s attentional capacity is one of the positive effects nature has on humans [6,13].

It could be expected from the theory of Ulrich et al. [5] that natural environments would reduce stress compared to built-up and noisy environments. Several studies focussing on physiological stress-releasing effects of natural compared to urban built areas found reductions in heart rates, pulse rates, salivary cortisol and blood pressures [4,5,14,15,16,17,18], supporting SRT [5]. Several field experiments found that, compared to urban street environments, forests provide higher physiological health benefits [14,15,16]. However, other studies could not observe different physiological effects on blood pressure, salivary cortisol, or heart rates as a result of visits to grey versus natural environments or stays in natural outdoor versus built indoor environments [1,19,20,21,22]. Lanki et al. [23] found no differences in the blood pressure of women as a result of stays in a forest, a park and the city centre. However, when they separated active (walking) from passive (seating) activities, they found that a visit to a forest was associated with lower systolic blood pressure compared to a visit to the city centre. Sonntag-Öström et al. [24] found a decreased diastolic blood pressure after visits to a forest compared to a busy urban street environment, while systolic blood pressure did not vary. In their review, Kondo et al. [25] summarized that most studies found lower heart rates in green spaces compared to built-up environments, while no associations between urban green space exposure and blood pressure were observed.

The length of exposure to natural environments seems to impact human health. Positive effects on physiological and psychological parameters become apparent even after a forest visit of only few minutes [17,21,26,27]. Longer stays seem to have additional positive effects [27,28,29]. Grazuleviciene et al. [30], for example, allocated participants to a 7-day controlled walking activity in a city park or urban street environment and found that the reduction of diastolic blood pressure and salivary cortisol was evident in the park group on the seventh day. However, other studies could not identify beneficial effects of time [31,32]. So far, health effects of a field experiment lasting several consecutive days with multiple exposures to green and blue environments in a mountain environment have not been explored.

Relationships between human wellbeing and mental health and the degree of naturalness have been analysed in several studies, mostly in the urban context and with partly contradicting outcomes. While Martens et al. [33] found that a forest with lower levels of structural variety increases wellbeing compared to a less managed forest, Carrus et al. [34] found that wellbeing increases with the level of naturalness. Wallner et al. [35] analysed the wellbeing and cognitive performance of pupils in different urban green spaces and found that this was almost always highest after the stay in the three green space types compared to the classroom situation. A sustained effect was only found for the most natural site. Similarly, concentration performance values were significantly higher after the pupils’ stay in green spaces for all sites. The highest increase of performance was found for the site with a medium degree of naturalness. Hansmann et al. [36] and Tyrväinen et al. [1] found only limited evidence that forests have a more positive influence on human health compared to designed city parks. Beil and Hanes [21] did not find differences in salivary cortisol and in self-reported stress for settings with different degrees of naturalness. Marselle et al. [8] also found no relationships between perceived naturalness and post-walk emotional well-being. Similarly, perceived health benefits did not differ between two mountain meadows with differently perceived degrees of naturalness [7] or between parkland, tended woodland and wild woods [9]. Several researchers [7,37] called for more research to produce a more robust evidence base for the relationships between degree of naturalness, landscape types and human health. Different understandings of naturalness in the studies make interpreting the relationship more difficult.

### 1.2. Health Benefits of Blue Environments on Humans

So far, little evidence exists for the physiological health benefits of blue spaces—i.e., aquatic environments such as rivers, lakes or oceans—compared to green or built environments [38]. In particular, perceived and measured health effects of stays at mountain rivers have rarely been documented. Ulrich et al. [5] found no differences in several physiological stress measures such as pulse transit time, spontaneous skin conductance, and muscle tension between respondents watching videos of a fast moving stream with sound levels of 63.5 dB and a natural green site with sound levels between 42 and 64 dB. Similarly, van Den Berg et al. [12] did not find differences in stress levels between respondents watching videos of natural landscapes with or without water, although several visual preference studies showed a higher preference of humans for blue, compared to green, spaces [39,40].

Recent field experiments also provided mixed findings about the restorative role of blue, compared to grey or green, environments. Frohmann et al. [41] compared the impact of a stay at a waterfall with that of a small forest and a rocky terrain in mountain areas on heart rate variability and found that vegetative relaxation was highest in the forest, while heart rates were most activated at the waterfall. Sonntag-Öström et al. [24] and Barton and Pretty [28] found that the presence of water was associated with greater improvements in mood compared to grey or green environments. In their field experiment, Triguero-Mas et al. [31] showed that people with indications of psychological distress reported lower mood disturbances and had favourable changes in heart rate variability indicators compared to the urban environment, while authors did not find differences in blood pressure. Gidlow et al. [42] found no differences in mood and cortisol levels between walking in residential, blue or green environments, while both blue and green environments provided greater restorative experiences and more cognitive benefits than the grey one.

### 1.3. Research Questions

In a direct response to the call for more research on the relationships between human health and environments with different degrees of naturalness [25,37], and the request for more field experiments [1,25], this study compared health effects of mountain meadows and a mountain river with an urban site of a depended sample of 22 participants. The research questions (RQ) were:

- *RQ 1:* Do managed and abandoned mountain meadows have different effects on the physiological (RQ 1a) and psychological (RQ 1b) health of participants?
- *RQ 2*: Do mountain meadows and a mountain river have more positive impacts on physiological (RQ 2a) and psychological (RQ 2b) health of participants compared to an urban site?
- *RQ 3*: Does a journey through the Alps lasting several days with a number of stays in green and blue environments have effects on the physiological and psychological health of participants?

## 2. Materials and Methods

### 2.1. Study Areas

Four study regions across the Alpine range from Austria to Switzerland were considered in the project: The City of Vienna, Long-Term Socio-economic and Ecosystem Research (LTSER)-Region Eisenwurzen in the province of Styria, Großes Walsertal UNESCO Biosphere Reserve in the province of Vorarlberg (all in Austria), and Val Müstair UNESCO Biosphere Reserve in Switzerland. The study was supported by the Earth-System-Sciences-Programme (ESS), Man and the Biosphere-Section, which requested mountain biosphere reserves and/or LTSER-regions in Austria and Switzerland as study sites. 

**Figure 1:**
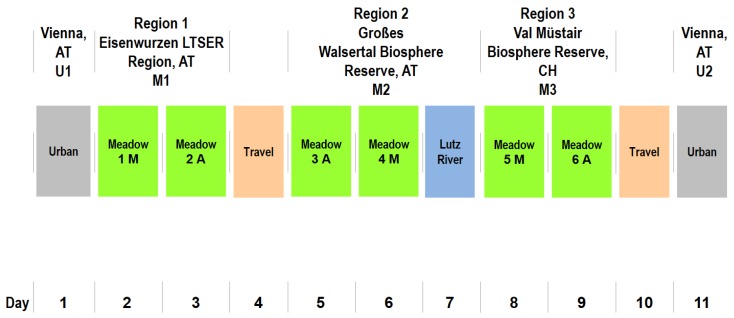
Sequence of exposures during the field trip (M = managed meadow; A = abandoned meadow).

**Figure 2:**
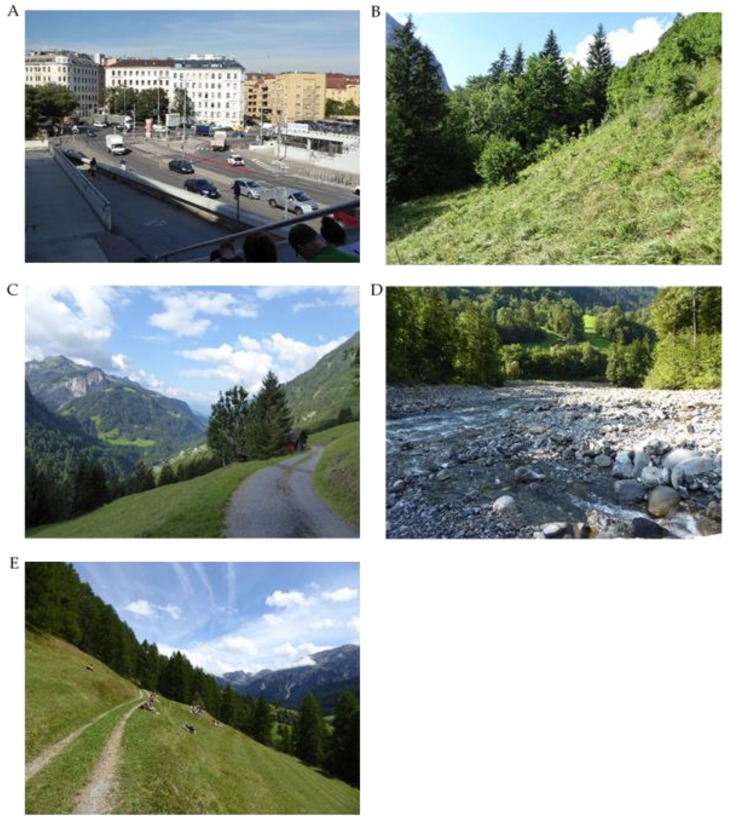
Photos of study sites (**A**) Vienna urban (U1, U2), (**B**) LTSER-region (M1), (**C**) Großes Walsertal (M2), (**D**) Lutz River, (**E**) Val Müstair (M3).

The 11-day field trip took place between 17 August and 27 August 2015 (Figure 1). The trip started with a measurement at the site in Vienna (U1), followed by a 2-day stay in the LTSER-region (M1). After one day of traveling to the west, 2.5 days were spent in the Großes Walsertal Biosphere Reserve (M2), followed by a 2-day stay in the Val Müstair Biosphere Reserve (M3). After one day of traveling back to Vienna, the trip concluded with a measurement at the same site in Vienna (U2) to test the effects of the stays in several natural environments in the mountains through a comparison between U1 and U2. Within each non-urban study region (M1–M3) an abandoned mountain meadow (unmanaged for at least 10 years) and a neighbouring extensively-managed (mowed once a year, no fertilizer use) mountain meadow were selected (Figure 2). All meadows were located on south-facing slopes. Abandoned meadows were characterised by higher grass, shrubs and initial stages of wood with few higher trees. Measurements at a mountain river were taken at the shoreline of the Lutz River in M2 (Figure 2). The urban site (U1, U2) was a broad stairway at the entrance area of a university in an inner urban area of Vienna (Figure 2). From this higher elevated point, participants observed a larger street crossing with heavy traffic use and noise.

### 2.2. Participants

A sample of 22 voluntary and healthy participants (12 females, 10 males), and of a fairly homogeneous age (age mean = 26.7, SD = 4.1, ranging from 22 to 36 years), was used for the field trip. Exclusion criteria were: smoking, heart diseases, medication for blood pressure, pregnancy, and age over 45 because a high age range most likely increases between-subject variations in physiological responses. The sample consisted of working people and students from various universities. Before the trip, participants filled in a baseline questionnaire asking them about their employment, health status, attitudes towards travelling in a group, nature orientation, and landscape preferences.

Participants were recruited on a voluntary basis by distributing flyers at different universities and research institutions and personal contacts. Participants were informed about the study and methods used, associated risks, and confidentiality issues. All subjects gave their informed consent for inclusion in writing before they participated in the study and for the collection and analysis of data. The study was conducted in accordance with the Declaration of Helsinki, and the study design was approved by the scientific advisory board of the ESS-programme (project identification code: 10470). Respondents received a small remuneration for their efforts. Each participant was assigned a code number, and participants used their code during the whole experiment. 

For an expected effect size (f) of 0.3, an alpha level of <0.05, and a power of 80%, the sample of 22 subjects was adequate to statistically determine relevant differences. Comparable field studies relied on similar numbers of participants [17,21,23,24,29,30,31,43].

The participants filled in a daily report every morning: Participants evaluated their actual health state, mental and physical constitution and their mood, as well as the quality of their sleep in the previous night. None of the participants fell ill during the field trip. Participants were not allowed to consume alcohol or undertake hard physical activities before each site visit.

We visited the study sites with the whole group. Several other field studies split their participants into smaller groups [17,23,43]. Consequently, each participant was exposed to the same environmental conditions during the field trip, with the exception of the last night, where participants were allowed to stay at home to provide a comparable condition with U1. Participants were advised to behave in the same way as the nights before. As participants were always in a group and under the supervision of the research team, the same activities were undertaken. During the course of the experiment, participants consumed similar food at the same times.

### 2.3. Survey Instruments

#### 2.3.1. Blood Pressure and Pulse Rates

Measurements at mountain meadows were conducted in rough and remote terrain. These circumstances required easy-to-use measuring instruments and non-invasive methods. To test physiological parameters of the cardiovascular system (pulse, systolic (SBP) and diastolic blood pressure (DBP)) in a practicable and reliable manner, self-inflating wrist blood pressure cuffs (boso medilife S) were used. Pulse, SBP and DBP were measured three times per study site. Before any physiological measurement participants were sitting quietly in upright position for at least 5 min, potentially eliminating activity-related effects. No talking during the measurement procedure was allowed. The third (most reliable) measurement of pulse and blood pressure was used for analyses. We did not consider potential offsets of the immune system [29] what is a limitation of the work.

#### 2.3.2. Questionnaire

The psychological health benefits (i.e., attention restoration, stress relief and wellbeing) were assessed using single-item 5-point answer scales. Participants were asked whether their stay at the study site had restored their attention (1 = very well, 5 = not at all), reduced their stress level (1 = very well, 5 = not at all), and changed their psychological wellbeing (1 = improved, 3 = unchanged, 5 = declined).

The questionnaire addressed perceptions of the scenic beauty of the surrounding landscape and of the study site itself, sound perceptions and awareness of background sound using 5-point answer scales. Landscape and site beauty were assessed by an answer scale which ranged from 1 = very high to 5 = very low. The answer scale of the perceived sound level ranged from 1 = quiet to 5 = very loud; perceived background sound ranged from 1 = very pleasant to 5 = very unpleasant. Participants had to rate the suitability of each site for recreation purposes (1 = very useful, 5 = absolutely not), and whether they would revisit this site for recreational purposes on a scale ranging from 1 = definitely yes to 5 = definitely not.

Psychological resilience was used to test whether the repeated exposure to several mountainous landscapes has an effect on perceived health. Psychological resilience is considered to be the ability of a human being to cope with extraordinary requirements and challenging situations in everyday life through personal and social, passed-on resources and to use it for individual development at the same time [44]. This psychological concept explains why some humans handle daily strains better and are less vulnerable than others. Measuring this ability helps to understand if staying in a natural environment over several days leads to an improvement in the resilience of the probands. The short version (RS 11) of the original “resilience-scale”, developed by Schumacher et al. [45] was used. The answer scale ranged from 1 = totally agree to 7 = totally disagree, with lower values indicating higher resilience. Based on a high internal consistency (Cronbach’s alpha >0.70) items were aggregated.

### 2.4. Procedure

The 22 participants visited all study sites in a standardised manner (Table 1). Each measurement day started at 8:30 a.m. and the experiment lasted 2.5 h. Each site visit strictly followed the same procedure (i.e., same time of the day; similar physical activity level, and length of stay) during the entire research trip. Pulse rate and blood pressure were measured four times per study site visit. Before departure at 8:30 a.m., participants measured their pulse rates and blood pressure [T1]. Participants arrived near the study site by bus, after a journey of between 25–30 min, and measured their pulse rates and blood pressure again [T2]. On arriving at the study site, after an easy 10-min walk in flat terrain or a short shuttle transport of about 10 min (M2), participants sat and observed the scenery for 15 min, after which they measured pulse rates and blood pressure again [T3]. Then they observed the scenery again for some minutes and filled in several survey forms, dealing with perceptions of sound, aesthetics, and perceived health benefits. Participants then walked back to the bus and measured pulse rates and blood pressure [T4] before returning to their accommodation by bus. We tried to level-out the order effect by rotating the meadow visits in terms of time depending on land-use intensity (managed/abandoned). For instance, in Areas M1 + M3 we visited the managed meadow at the first day and the abandoned one at the next day, while in Area M2 the abandoned meadow was visited. An order effect may exist for the regions and the river. Psychological resilience was asked two times per day on travel days; before the first measurement and in the late afternoon.

### 2.5. Environmental Data

Environmental data were collected following Lanki et al. [23]. During the measurements at each site, noise levels were permanently monitored using a standard noise measurement device (SL-451, Voltcraft, Hirschau, Germany) recording noise levels at a 1-s interval. Based on 30 min of observation, an average noise level was calculated (Table 2). The average noise level was 49.9 dB(A) (SD = 10.6) and differed significantly between the sites (*F* = 22,863, *p* < 0.001). Noise levels were highest at the urban site (U1: 61.1, U2: 63.3 dB(A)) and at the mountain river with 66.6 dB(A). Noise levels at the meadows ranged between 40.6 and 45.2 dB(A) with the lowest noise levels at M2. Noise levels differed significantly among U1 and U2 (*t* = 21.252, *p* < 0.001) and among the meadow sites (*F* = 1950.0, *p* < 0.001) with M2 being the quietest site.

In addition, human observers counted the number of visitors and cars directly passing by. However, visitor and car numbers, ranging between 0 and 2 visitors and/or cars, were negligible at the natural sites. Heavy traffic could be observed at a distance of about 100 m from the urban site, and much further away from M1 and M3, at a road at the distant valley bottom. Weather observations were carried out by recording ambient temperatures, relative humidity and weather conditions (Table 2). Average temperature at the sites was 19.6 °C (SD = 2.5) and average relative humidity was 63.6% (SD = 10.0). On most days, the weather wFUas sunny to cloudy, with little wind, except for M3 where there was some drizzling rainfall on the first day of measurement.

### 2.6. Analyses

We used General Linear Models with repeated measures to analyse differences in pulse rates, blood pressures and perceived health effects, noise and landscape perceptions between the study sites. Time and site for pulse and blood pressure analyses, and site for psychological measures were the within subject factors. ANOVAs with repeated measures are susceptible to the violation of the assumption of sphericity. We therefore used Mauchly’s tests of sphericity to evaluate whether the variances of the differences were equal. If violations of sphericity did occur, we used the Greenhouse-Geisser correction factor from analyses that encompassed more than two measures to produce a more valid F-value, as suggested by Rasch et al. [46]. Bonferroni post-hoc contrasts were used to identify differences between the visits. Pearson correlations were used to analyse relationships between SBP, DBP and pulse, and other items. SBP and DBP were highly correlated at all sites and measurements (*r* > 0.607), while only two moderate significant correlations were observed between DBP and pulse rates at T1 (U1, river). No correlations were found between temperature and pulse rates, SBP and DBP and between measured noise levels and pulse rates, SBP and DBP. A significance level of *p* ≤ 0.05 was chosen.

## 3. Results

### 3.1. Physiological Parameters

In response to RQ 1a, we first tested whether there are differences in cardiovascular parameters (pulse rates, SBP and DBP) between managed and abandoned meadows. As there were no significant differences between managed and abandoned meadows, while differences between the sites (M1, M2, M3) were found, the measured values of managed and abandoned meadows per site were aggregated (Pulse *F*(2, 22) = 3.597, *p* = 0.072, *η*^2^ = 0.146; SBP *F*(2, 22) = 0.312, *p* = 0.583, *η*^2^ = 0.015; DBP *F*(2, 22) = 1.810, *p* = 0.193, *η*^2^ = 0.079).

**Figure 3:**
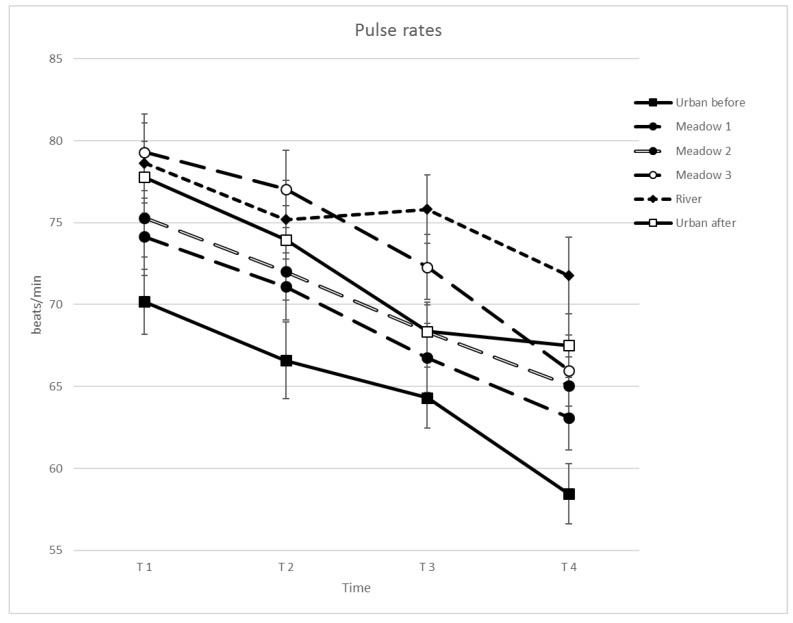
Mean pulse rate per site and time (*N* = 22).

In response to RQ 2a, pulse rates, SBP and DBP were compared between M1, M2 and M3, the river, and U1 and U2. Significant differences for pulse rates were obtained for site (*F*(5, 22) = 16.538, *p* = 0.000, *η*^2^ = 0.441), time of measurement (*F*(3, 22) = 85.874, *p* = 0.000, *η*^2^ = 0.804) and interactions between site and time (*F*(15, 22) = 3.680, *p* = 0.002, *η*^2^ = 0.149). Highest average pulse rates were observed at the river, followed by M3. Lowest pulse rates were measured at U1 and M1 (Table 3; Figure 3). Most differences in pulse were observed for U1, differing with all other sites except for M1, while no site differences were found for U2. Among the meadows, differences were found between M1 and M3 and between M2 and M3. The river differed from U1, M1 and M2. Differences in time were observed between all measurements (T1–T4). There was a significant and continuous decrease in pulse rate with time (T1–T4) at all sites except for the river. Most interactions were found for T3 and T4 and for U1 and the river. Pulse rates at U1 were lower at T1 and T2 compared to M3 and the river; at T3, lower compared to M3 and the river; and lower against all other sites at T4. Pulse rates measured at the river were higher at T3 and T4 compared to all other sites; except for T4 with no differences compared to U2.

**Figure 4:**
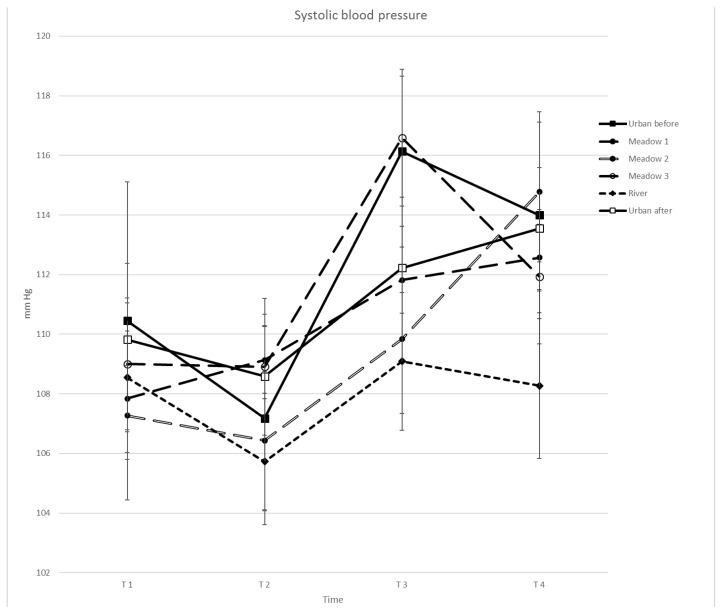
Mean systolic blood pressure per site and time (*N* = 22).

**Figure 5:**
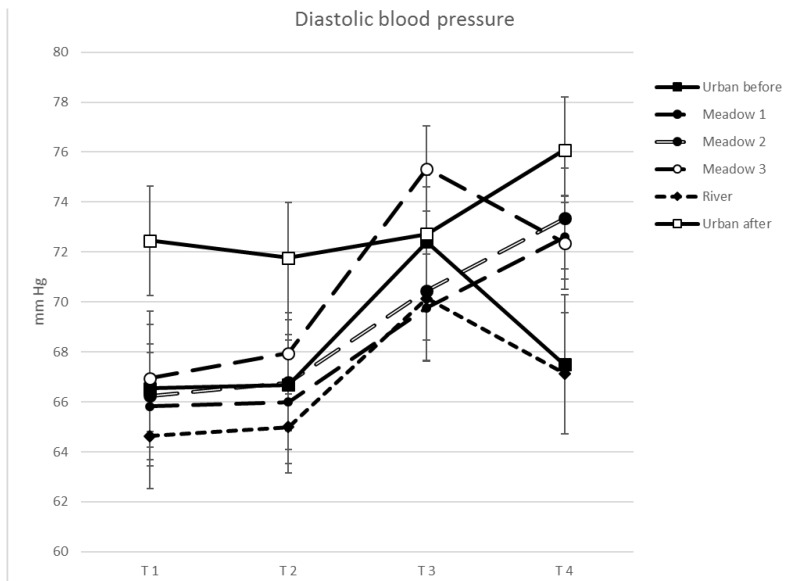
Mean diastolic blood pressure per site and time (*N* = 22).

Differences across sites were obtained for DBP (*F*(5, 22) = 5.880, *p* = 0.002, *η*^2^ = 0.219) but not for SBP (*F*(5, 22) = 2.334, *p* = 0.097, *η*^2^ = 0.100). Significant effects of time of measurement for SBP (*F*(3, 22) = 25.041, *p* = 0.000, *η*^2^ = 0.544) and DBP (*F*(3, 22) = 24.281, *p* = 0.000, *η*^2^ = 0.536) and significant interactions between sites and time for DBP (*F*(15, 22) = 2.488, *p* = 0.018, *η*^2^ = 0.106) but not for SBP (*F*(15, 22) = 1.800, *p* = 0.139, *η*^2^ = 0.079) were found. Increases in SBP and DBP were observed at all sites with differences between T1 and T3 and T4, and between T2 and T3 and T4, but not between T1 and T2 and between T3 and T4 (Table 4; Figure 4 and Figure 5). U2 was significantly higher in DBP compared to M2 and the river. DBP was lower at the river compared to M3. A significant interaction for DBP at T1 was found with U2 being higher than M2. At T2, U2 was higher than at the river. At T3, M3 was higher than the river, M1 and M2. At T4, U2 and M2 were higher than at the river.

Finally, we tested the effects of the journey on physiological parameters by comparing U1 with U2 (RQ 3). Pulse rates and DBP were higher at U2 compared to U1 (Pulse *F*(1, 22) = 19.267, *p* = 0.000, *η*^2^ = 0.478; DBP *F*(1, 22) = 5.512, *p* = 0.029, *η*^2^ = 0.208), while no differences in SBP were found (*F*(1, 22) = 0.156, *p* = 0.697, *η*^2^ = 0.007). Significant interactions were only found for DBP at T4 (*F*(3, 22) = 2.898, *p* = 0.046, *η*^2^ = 0.121), with higher values for U2 compared to U1.

### 3.2. Health, Sound and Landscape Perceptions and Psychological Resilience

In response to RQ 1b, we tested whether there are differences in perceived health benefits (stress reduction, attention restoration and wellbeing) between managed and abandoned meadows. No differences were found for stress reduction, attention restoration and wellbeing between managed and abandoned meadows (stress reduction: *F*(1, 22) = 0.129, *p* = 0.723, *η*^2^ = 0.006; attention restoration: *F*(1, 22) = 0.272, *p* = 0.114, *η*^2^ = 0.115; wellbeing: *F*(1, 22) = 0.071, *p* = 0.792, *η*^2^ = 0.003). Therefore, the values of managed and abandoned meadows per site were aggregated.

In response to RQ 2b, differences in perceived health benefits were found for attention restoration, stress reduction and wellbeing across sites (*p* < 0.001). Bonferroni post-hoc contrasts found that participants reported the same and highest positive effects of their stays at M2 and the river for attention restoration, stress reduction and wellbeing (Table 5 and Table 6). M1 and M3 received the same evaluations but were significantly lower than M2 and the river. U1 and U2 received the same and lowest scores of all sites. For wellbeing, M1 did not differ from M2, M3 and the river, and M2 did not differ from M3 for attention restoration.

Differences between the sites were also found for the perceived suitability for recreation (*p* < 0.001) and the probability of a revisit for recreational purposes (*p* < 0.001). The river and M2 were similarly perceived as most suitable for recreation, followed by M1 and M3, and finally the urban site (Table 5 and Table 6). The same answer patterns were observed for the probability of a revisit for recreational purposes. It was highest and the same for the river and M2, followed by the other meadows. The respondents would not consider the urban site for a revisit.

Differences were also observed for acoustic perceptions (*p* < 0.001) and perceptions of the background sound (*p* < 0.001) (Table 5 and Table 6). Overall, perceptions of sound were highest and similar at U1, U2, the river, and M1, while lowest for M2. Different answer patterns were received for the perceptions of the background noise. M2 was perceived as the site with the lowest background sound, followed by the river. Both sites differ from all sites. Perceived background sound at U1, M1 and M3 was the same. Background sound was highest at U2.

Respondents perceived M2 as surrounded by the most beautiful landscape, followed by the other meadows and the river that all received the same scores (Table 5 and Table 6). The beauty of the urban site was lowest. The river, M2 and M3 were the sites with the highest beauty, followed by M1, which differed from the river but not from M2 and M3.

**Figure 6:**
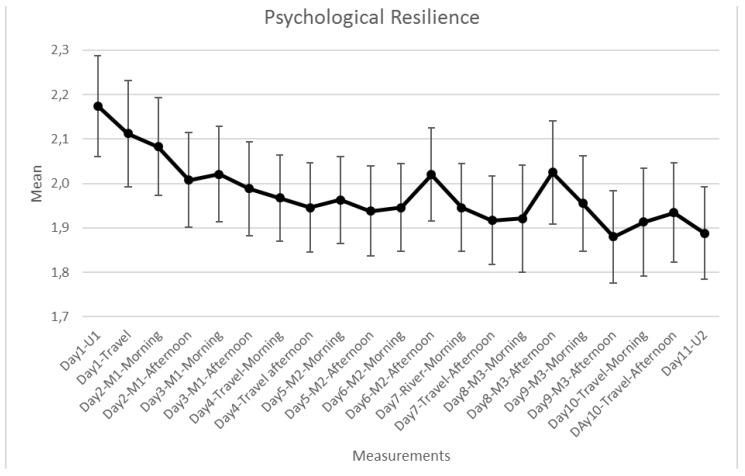
Psychological resilience of participants during the field trip (*N* = 22; Answer scale 1 = totally agree to 7 = totally disagree).

In response to RQ 3, no differences were found between U1 and U2 for all items reported above. There was a significant increase in psychological resilience (*F*(20, 22) = 2.724, *p* = 0.023, *η*^2^ = 0.115; Figure 6) as the field trip progressed. Significant differences in psychological resilience appeared after the second day at M1 compared to the start of the field trip at U1 (*F*(1, 22) = 5.783, *p* = 0.025, *η*^2^ = 0.216). A further significant decrease with time was not observed. There were no differences in psychological resilience between the morning and afternoon measurements (*F*(1, 22) = 0.021, *p* = 0.886, *η*^2^ = 0.001).

## 4. Discussion

To the best of our knowledge, this is one of the first studies to compare the effects of short stays on measured and perceived human health among differently managed mountain meadows, at a mountain river and of an urban site. This study found differences and commonalities between the sites for physiological health measures and perceived health benefits.

### 4.1. Physiological Effects

No differences in measured health effects were observed between managed and abandoned meadows indicating that health effects appear to be independent of the degree of naturalness. This confirms research showing no consistent patterns of physiological parameters such as blood pressure or salivary cortisol in response to different levels of naturalness within green environments [1,19,20,21,25]. Blood pressure and pulse rate results indicate that the region is more important than the degree of naturalness or the management of the meadows.

Across all site visits, pulse rates decreased more or less linearly except for those measured at the river. This indicates that all sites—independent of whether the location was grey or green—had a calming and sustained positive effect on participants after the stay. Most previous research has also observed a decrease in pulse or heart rates after stays in semi-natural areas [4,23]. Compared to several previous studies comparing grey with green sites [15,16,17,43], this study did not measure health effects at a street. Participants observed the traffic from a distant and safe location without any direct traffic causing impacts such as vibrations. This study suggests that noisy grey sites where participants are not directly exposed to streets, can also reduce pulse rates. While previous restorative research focused on the impacts of green versus built environments, future research might investigate the effects on human health of different qualities of grey environments.

In contrast to the grey and green sites, a slight increase in pulse rates was observed during the stay at the river. The measured and perceived high sound levels may not be an explanation because we observed a decrease in pulse rate at the grey site (U1, U2) with similar measured and perceived sound levels. The riverside results may indicate an activation of the cardiovascular system [41] or increased vitality [47]. In contrast, Ulrich et al. [5] could not identify differences in physiological stress measures between respondents watching videos of a noisy fast-moving stream and a quieter natural green site. Summarizing, this study found positive effects of visits on pulse rates but could not find differences between green and grey environments. The outstanding role of the mountain river for pulse rates remains to be further explored.

Site differences were observed for DBP, and, in contrast to pulse results, an increase in SBP and DBP across all study sites with time and no sustained effect. Several studies found a stronger decrease in blood pressure in natural or semi-natural, compared to built-up, environments [5,14,15,16,18,19,23], while other authors could not find such a pattern [19,20,23]. Lanki et al. [23] also observed a marginal increase in SBP, as well as a significant one for DBP, after sitting in grey and green environments for 15 min, but the increase in SBP was lower in the forest than in the city. Our field study involved non-stressed participants—although any potential group effects are unknown—and requested very little physical activity, compared to many other studies where participants either walked in the environments [14,15,16,17,27] or exercised on a treadmill [18]. The passive activity may have influenced blood pressure differently as found by Lanki et al. [23]. However, Park et al. [16] found no differences in systolic and diastolic blood pressures between walking and viewing.

The effects of a short-term stay, either in grey, blue or green sites on blood pressure were marginally different. Study results show that each site visit had a similar impact on systolic blood pressure, while it observed differences in main effects of site and interactions for DBP. The increase in systolic and diastolic blood pressure, and the missing sustained effect (no decrease) indicate no calming effects, not supporting the SRT [5]. Only the overall higher DBP and the missing sustained effect (T4) at U2 for DBP are indications that natural environments, in particular the river, are more beneficial for human health. However, we could not observe this effect for U1. Although highly correlated, and similar time effects were observed, SBP and DBP did not work conformally, as found, for example, by Park et al. [16]. Lanki et al. [23] and Sonntag-Öström et al. [24] also observed different responses of SBP and DBP associated with visits to different sites. Because of their different effects, both, SBP and DBP, as well as pulse rate, might be used for assessing health effects of the cardiovascular system.

### 4.2. Psychological Effects

In accordance with the results of the pulse and blood measurements, perceived health benefits (attention restoration, wellbeing, and stress reduction) did not differ between managed and unmanaged meadows. Few previous studies have compared similar land use or vegetation types, such as forests or meadows, with different degrees of naturalness [7,9,33]. This study compared diverse mountain meadow regions with varying degrees of naturalness and study results confirm those studies that have not found differences in health effects depending on the degree of perceived or actual naturalness [7,8,9] but contradict others [33]. This study shows that open (managed meadows) and semi-open (unmanaged meadows) landscapes provided the same physiological health benefits.

All natural sites provided higher self-reported health benefits to participants compared to the urban one (i.e., attention restoration, wellbeing and stress reduction). This finding confirms the assumptions of the ART and the SRT [5,6], assuming that a stay in natural environments is more beneficial for mental and physiological restoration.

Participants perceived the river as one of the most restorative sites, although measured and perceived noise levels were very high and the pulse rate did not decrease. The positive perception of the river was also confirmed with the high recreational value participants assigned the river, the high beauty of the river landscape itself and a low diastolic blood pressure. That blue environments are perceived as rather restorative sites has been confirmed in other studies [38,40]. However, few—if any—studies have investigated the restorative effects of a mountain river. This study shows that, although the river site did not provide many views over the Alpine scenery, a loud mountain river is perceived to be similarly—or even more—beneficial to human health than mountain meadows with their views on the mountainous scenery. M2 was equally perceived as beneficial for participants’ mental and physical health and as recreation site. M2 was the most remote of the study sites with the lowest measured and perceived noise levels and perceived highest beauty of all the sites. Physiological parameters could neither support nor contradict the reported health perceptions of the participants. For DBP, even a further increase was found at T4. Maybe the beauty of the site evoked an arousal effect.

Summarizing, the river and an alpine mountain meadow provided the highest perceived health benefits for respondents. However, psychological and physiological results do not suggest convergent validity, although they do not directly contradict each other. Psychological measures provided a more consistent picture about perceived health effects, and found differences between and among the natural and urban sites. The results of the physiological measures were inconsistent regarding the question of which site provides the highest health benefits to participants. This study shows that mountain meadow sites differ in their perceived and measured health effects. Although the river and M2 provided different landscape qualities (measured and perceived noise, landscape beauty), their effects on human health benefits were equally rated by participants.

This study also suggests that a journey through the Alps lasting several days with a number of exposures to green and blue environments has positive effects on psychological resilience. The positive effect was already achieved after two days. The journey also affected pulse rates and DBP but did not change the health perceptions of participants, when comparing U1 and U2. It seems that the urban environment had more negative impacts on the physiological health of the participants after the journey than before. Whether participants became more sensitive to urban environmental conditions during their stay in the Alps, or whether other factors caused this increase, has to be left to further research. Additional field studies across different alpine land-uses and altitudes, and the use of other research methods investigating, for example, heart rate variability or using cortisol measures [1,5,41,43] may provide a deeper understanding of the health effects of mountain environments. It is a limitation of the study, that we did not rely on permanent recording of pulse, SBP and DBP during field visits. However, this was not feasible within our study design. Other limitations of the study are the lack of a control group, the fact that the participants were volunteers, and potentially indicate a self-selection bias, and that—compared to other studies—participants were not stressed before the measurements [9,22].

## 5. Conclusions

This study found that participants perceived natural environments as providing more health benefits to humans than the urban site. However, visits to both natural and urban environments were associated with a decrease in pulse rate and an increase in blood pressure with only marginal differences between green and urban sites. Although an increase in pulse rates was observed at the noisiest site in the study—the mountain river—participants assigned it with a high restorative potential. Its restorative potential was equal to the meadows of the Großes Walsertal Biosphere Reserve (M2), which were perceived as the most beautiful and quietest sites, indicating that measured and perceived differences in landscape qualities can even result in similar ratings of health benefits. Differences in perceived or measured health benefits between meadows with varying degree of naturalness were not found. The different results of cardiovascular parameters and psychological measures may indicate that future studies employ several parameters for measuring health effects for a better understanding of the nature-health relationship.

Study results may indicate that restorative sites can be found to some degree in urban built-up and, in particular, mountainous areas. Mountain rivers and remote alpine meadows, independent of their degree of naturalness, seem to provide health benefits, and may have a potential for the development of health-specific offers.

## Figures and Tables

**Table 1:** Detailed daily data collection procedure.

Daytime (Approximately)	Procedure	Activity	Measurement
8:30 a.m.	First measurement (T1), then departure to study site by bus	Sitting	Pulse rate Blood pressure Psychological resilience
9:10 a.m.	Arrival at the bus parking lot near the study site, followed by second measurement (T2)	Sitting	Pulse rate Blood pressure
9:25 a.m.	Walk (drive) to the study site	Easy walking or sitting (shuttle transport at M2)	
9:35 a.m.	Stay at the study site	Sitting & watching	
9:50 a.m.	Third measurement (T3)	Sitting	Pulse rate Blood pressure Questionnaire
10:15 a.m.	Departure from study site	Easy walking or sitting (shuttle transport at M2)	
10:25 a.m.	Stay at the bus parking lot	Sitting	
10:30 a.m.	Fourth measurement (T4), then departure by bus	Sitting	Pulse rate Blood pressure

**Table 2:** Environmental data per study site and day.

Means	U1 Day 1	M1 Days 2/3	M2 Days 5/6	River Day 7	M3 Days 8/9	U2 Day 11
Noise level (LAeq [dBA])	61.1	45.2/41.2	40.6/41.1	66.6	45.2/44.3	63.4
Temperature [°C]	20.9	22.0/20.1	20.6/15.9	22.5	19.8/15.1	19.9
Humidity [%]	76%	60%/63%	55%/58%	50%	78%/74%	58%
Weather conditions ^a^	2	1/2	1/1	1	3/1	1

^a^ 1 = sun and clouds; 2 = cloudy; 3 = light drizzling rain.

**Table 3:** Means and SD (in brackets) of pulse rates per study site (*N* = 22).

Pulse Rate (Mean)	U1	M1	M2	River	M3	U2	Average
T1	70.2 (9.3)	74.1 (11.1)	75.3 (11.3)	79.3 (10.9)	78.6 (11.4)	77.8 (10.2)	75.9 (9.6)
T2	66.6 (11.0)	71.1 (9.6)	72.0 (8.2)	77.0 (11.0)	75.2 (11.2)	74.0 (8.3)	72.6 (8.7)
T3	64.3 (8.8)	66.8 (9.9)	68.3 (7.6)	72.3 (9.4)	75.8 (9.8)	68.4 (8.4)	69.3 (7.7)
T4	58.5 (8.6)	63.1 (9.1)	65.1 (8.1)	66.0 (10.2)	71.8 (10.9)	67.5 (9.0)	65.3 (8.1)
Average	64.9 (9.0)	68.8 (9.7)	70.2 (8.2)	73.7 (10.0)	75.4 (10.2)	71.9 (8.2)	

**Table 4:** Means and SD (in brackets) of systolic (SBP) and diastolic (DBP) blood pressure per study site (*N* = 22).

**SBP**	**U1**	**M1**	**M2**	**M3**	**River**	**U2**	**Average**
T1	110.5 (21.8)	107.8 (11.9)	107.3 (13.3)	109.0 (10.4)	108.5 (11.8)	109.8 (12.0)	108.8 (11.3)
T2	107.2 (14.6)	109.1 (12.7)	106.4 (10.9)	108.9 (10.8)	105.7 (9.9)	108.6 (9.8)	107.7 (10.1)
T3	116.1 (11.8)	111.8 (11.4)	109.8 (11.8)	116.6 (10.8)	109.1 (10.9)	112.2 (11.2)	112.6 (10.1)
T4	114.0 (16.3)	112.6 (11.2)	114.8 (11.0)	111.9 (10.6)	108.3 (11.5)	113.5 (9.6)	112.5 (10.0)
Average	111.9 (13.6)	110.3 (11.2)	109.6 (11.4)	111.6 (10.1)	107.9 (9.9)	111.0 (9.4)	
**DBP**							
T1	66.5 (14.5)	65.8 (10.0)	66.3 (9.7)	67.0 (10.1)	64.6 (9.9)	72.5 (10.2)	67.1 (8.9)
T2	66.7 (12.2)	66.0 (11.5)	66.8 (9.0)	68.0 (7.6)	65.0 (8.7)	71.8 (10.3)	67.4 (8.5)
T3	72.4 (10.3)	69.8 (10.1)	70.5 (9.3)	75.3 (8.1)	70.1 (11.6)	72.7 (11.0)	71.8 (8.6)
T4	67.5 (13.1)	72.6 (7.8)	73.3 (9.5)	72.4 (8.7)	67.1 (11.3)	76.1 (9.9)	71.5 (8.3)
Average	68.3 (10.7)	68.5 (9.3)	69.2 (8.9)	70.7 (8.0)	66.7 (9.2)	73.3 (9.0)	

**Table 5:** Means of perceived health effects, recreation, noise and landscape beauty per study site (*N* = 22).

Items (Mean)	U1	M1	M2	M3	River	U2	ANOVA Repeated Measures
**Health benefits and recreation**							
Attention restoration	4.05	2.41	1.70	2.18	1.73	3.91	*F* = 49.890; *p* < 0.001 *η*^2^ = 0.704
Stress reduction	3.91	2.27	1.52	2.12	1.55	3.77	*F* = 48.532; *p* < 0.001 *η*^2^ = 0.698
Wellbeing	3.33	2.00	1.50	2.02	1.48	3.29	*F* = 32.587; *p* < 0.001 *η*^2^ = 0.620
Suitability for recreation	4.73	2.86	1.80	2.66	1.77	4.45	*F* = 92.382; *p* < 0.001 *η*^2^ = 0.815
Would you revisit this site for recreational purposes?	4.55	3.16	2.00	2.72	1.55	4.32	*F* = 70.619; *p* < 0.001 *η*^2^ = 0.771
Perceived sound level	3.95	3.64	2.11	3.36	4.18	4.32	*F* = 35.817; *p* < 0.001 *η*^2^ = 0.630
Perceived background sound	3.64	3.57	1.41	3.45	2.14	4.36	*F* = 54.953; *p* < 0.001 *η*^2^ = 0.724
Surrounding landscape beauty	3.82	1.63	1.11	1.66	1.64	3.82	*F* = 77.735; *p* < 0.001 *η*^2^ = 0.787
Study site beauty	4.05	2.41	2.14	2.14	1.64	4.14	*F* = 58.551; *p* < 0.001 *η*^2^ = 0.736

Health benefits: attention restoration: 1 = very well, 5 = not at all; stress reduction: 1 = very well, 5 = not at all; Psychological wellbeing: 1 = improved, 3 = not changed, 5 = declined; Perceived sound level: 1 = very quiet, 5 = very loud. Perceived background sound: 1 = very pleasant, 5 = very unpleasant; Perceived meadow/landscape beauty: 1 = very high, 5 = very low; Suitability for recreation: 1 = very useful, 5 = absolutely not; Would you revisit this site for recreational purposes: 1 = definitely yes, 5 = absolutely not.

**Table 6:** Study sites differences between perceptions of health effects and landscape using Bonferroni post-hoc tests (*N* = 22).

	Sites	AR	SR	WB	REC	REV	PS	PBS	LB	SB
U1	M1	*	*	*	*	*			*	*
	M2	*	*	*	*	*	*	*	*	*
	M3	*	*	*	*	*	*		*	*
	River	*	*	*	*	*		*	*	*
	U2							*		
M1	U1	*	*	*	*	*			*	*
	M2	*	*		*	*	*	*	*	
	M3									
	River	*	*		*	*		*		*
	U2	*	*	*	*	*		*	*	*
M2	U1	*	*	*	*	*	*	*	*	*
	M1	*	*		*	*	*	*	*	
	M3		*	*	*	*	*	*	*	
	River						*	*	*	
	U2	*	*	*	*	*	*	*	*	*
M3	U1	*	*	*	*	*	*		*	*
	M1									
	M2		*	*	*	*	*	*	*	
	River	*	*	*	*	*		*		
	U2	*	*	*	*	*	*	*	*	*
River	U1	*	*	*	*	*		*	*	*
	M1	*	*		*	*		*		*
	M2						*	*	*	
	M3	*	*	*	*	*		*		
	U2	*	*	*	*	*		*	*	*
U2	U1							*		
	M1	*	*	*	*	*		*	*	*
	M2	*	*	*	*	*	*	*	*	*
	M3	*	*	*	*	*	*	*	*	*
	River	*	*	*	*	*		*	*	*

AR = Attention restoration; SR = Stress reduction; WB = Well-being; REC = Suitability for recreation; REV = Revisit this site for recreational purposes; PS = Perceived sound level; PBS = Perceived background sound; LB = Surrounding Landscape beauty; SB = Study site beauty; * differences between sites at *p* < 0.05.
